# Antihypertensive therapy during pregnancy: the prescription pattern in Italy

**DOI:** 10.3389/fphar.2024.1370797

**Published:** 2024-08-30

**Authors:** Anna Locatelli, Nicolò Bellante, Gianluca Donatiello, Filomena Fortinguerra, Valeria Belleudi, Francesca R. Poggi, Serena Perna, Francesco Trotta

**Affiliations:** ^1^ School of Medicine and Surgery, University of Milano-Bicocca, Monza, Italy; ^2^ Italian Medicines Agency (AIFA), Rome, Italy; ^3^ Departement of Epidemiology, Lazio Regional Health Service, ASL Roma 1, Rome, Lazio, Italy

**Keywords:** hypertension, pregnancy, antihypertensives, preeclampsia, prescription pattern

## Abstract

Drug use during pregnancy should be evidence-based and favor the safest and most appropriate prescription. The Italian Medicines Agency (AIFA) coordinates a network focusing on monitoring medication use in pregnancy. Hypertensive disorders are common medical complication of pregnancy and antihypertensive therapy is prescribed to reduce the risk of adverse feto-maternal complications. The objective of this study is to highlight the prescription pattern of antihypertensive drugs before pregnancy, during pregnancy and in the postpartum period in Italy and to evaluate their use with a specific attention to the prescription pattern of drugs considered safe during pregnancy. A multi-database cross-sectional population study using a Common Data Model (CDM) was performed. We selected all women aged 15–49 years living in eight Italian regions who gave birth in hospital between 1 April 2016 and 31 March 2018. In a cohort of 449.012 women, corresponding to 59% of Italian deliveries occurred in the study period, the prevalence of prescription of antihypertensive drugs in the pre-conceptional period was 1.2%, in pregnancy 2.0% and in the postpartum period 2.9%. Beta-blockers were the most prescribed drugs before pregnancy (0.28%–0.30%). Calcium channel blockers were the most prescribed drugs during pregnancy, with a prevalence of 0.23%, 0.33%, 0.75% in each trimester. Alfa-2-adrenergic receptor agonists were the second most prescribed during pregnancy with a prevalence of 0.16%, 0.26% and 0.55% in each trimester. The prescription of drugs contraindicated during pregnancy was below 0.5%. Only a small percentage of women switched from a contraindicated drug to a drug compatible with pregnancy. The analysis showed little variability between the different Italian regions. In general, the prescription of antihypertensive drugs in the Italian Mom-Network is coherent with the drugs compatible with pregnancy.

## Introduction

Hypertensive disorders are the most common medical complication of pregnancy and represent one of the major causes of maternal and perinatal morbidity and mortality worldwide (Borghi et al., 2013; Bortolotto et al., 2018; Hitti et al., 2018; Lodi et al., 2018; Nzelu et al., 2018; AIPE Associazione Italiana Preeclampsia, 2020).

High blood pressure may be associated with other complications, among which preeclampsia is the most common (Duley et al., 2006).

Due to the increasing age of women at childbirth, obesity and other frequent chronic pathologies, hypertensive disorders represent a severe global public health issue, being among the leading causes of maternal and perinatal mortality and morbidity worldwide and in Italy. In pregnancy the prevalence of chronic or pre-existing hypertension, gestational hypertension and preeclampsia is 5.2%–8.2%, 1.8%–4.4% and 0.2%–9.2%, respectively (Khan et al., 2006; Donati et al., 2012; Say et al., 2014; Lean et al., 2017; Ukah et al., 2018).

An association between maternal complications and severity of hypertensive disease was reported (Lean et al., 2017), as well as an increased risk of preterm birth and low birth weight (Macdonald-Wallis et al., 2014; Seely and Ecker, 2014; Macdonald-Wallis et al., 2012). Rates of preterm delivery are higher than general population among women with chronic hypertension ranging from 12% to 34%, and higher in women with severe hypertension ranging from 62% to 70% (Seely and Ecker, 2014).

Antihypertensive therapy is prescribed to treat chronic and gestational hypertension, and preeclampsia to optimize the gestational age at delivery, thus reducing the risk of prematurity and to prevent adverse feto-maternal complications, in particular placental abruption and cerebral events (Ukah et al., 2018). All antihypertensive drugs cross the placenta and the need to reduce fetal exposure to medications that may have adverse effects on fetus should be always considered (Sinkey et al., 2020; Tsakiridis et al., 2021).

However, a considerable paucity of data exists from randomized controlled trials to guide choice of antihypertensive agent for chronic hypertension in pregnancy (Webster et al., 2017; Fitton et al., 2017). The antihypertensive drugs which can be safely prescribed during pregnancy are methyldopa, labetalol and nifedipine (Gruppo di Studio Ipertensione in Gravidanza, 1998; Bortolus et al., 2000; National Institute for Health and Care Excellence UK, 2019).

On the contrary, Angiotensin Converting Enzyme (ACE) inhibitors and Angiotensin Receptor Blockers (ARBs) should be avoided during pregnancy due to a higher incidence of cardiovascular and central nervous system congenital malformations in fetus associated to the maternal use of these drugs (Magee and von Dadelszen, 2013; Fu et al., 2021). A recent Cochrane review concludes that there is not enough evidence to recommend a specific drug over others (Abalos et al., 2018). The most recent international guidelines suggest choosing between the different antihypertensive drugs according to clinician’s familiarity, patient preference and drug’s side effects (Webster et al., 2017; National Institute for Health and Care Excellence UK, 2019; Gestational Hypertension and Preeclampsia, 2020; Magee et al., 2022).

In 2015 and in 2022 the CHIPS and the CHAP randomized controlled trials were published (Magee et al., 2016; Tita et al., 2022). The CHIP trial aimed to evaluate the effects of less-tight versus tight control of hypertension during pregnancy both in patients with chronic hypertension and with gestational hypertension. The CHAP trial evaluated the safety and the benefits of treating mild chronic hypertension during pregnancy. The most prescribed drugs in the CHIPS trial were labetalol (66.9% and 67.3% in the less-tight and in the tight control groups, respectively), followed by alpha-methyldopa (43.5 vs. 40.3%) and nifedipine (31.8 vs. 30.1%). Similar results were observed in the CHAP trial.

In Italy, population-based studies on antihypertensive drug use in pregnancy are not recent and limited to single regional experience (Gagne et al., 2008; Ventura et al., 2018). In this perspective, the Italian Medicines Agency (AIFA–Agenzia Italiana del Farmaco) has promoted the creation of a network, the so-called MoM-Net (Monitoring Medication Use During Pregnancy - Network), aiming to monitoring the appropriate use of different classes of drugs in pregnancy, analyzing and integrating the data from different regional health databases.

The objective of this paper is to highlight the prescription pattern of antihypertensive drugs in Italy and evaluate its appropriateness to promote consequent intervention to improve clinical practice.

## Methods

### Data sources

We performed a multi-database cross-sectional population study using a Common Data Model (CDM. The data sources used to identify the study population were:

• The Regional Birth Registry (Certificato di Assistenza al Parto, CeDAP) which contains details about the sociodemographic aspects of mothers, pregnancy characteristics, and information about newborns.
• The Demographic Database, which is an administrative record that gathers information on individuals registered in the regional healthcare system.
• The Drug prescription database which encompasses details about regional prescriptions reimbursed by the Italian National Healthcare Service (NHS). This includes information such as the date of dispensing, number of packages, active ingredients, and brand.

The data sources can be interconnected via a distinct anonymous personal identification code at the regional level. The study employed an analytical method based on a CDM developed by the Lazio region, which guided the analysis of the data (Gini et al., 2020; Schneeweiss et al., 2020). Materials and methods were reported extensively in Belleudi et al. (2021).

### Analyses

The analytical approach facilitated the identification of women aged 15–49 residing in eight Italian regions (Lombardy, Veneto, Emilia Romagna, Tuscany, Umbria, Lazio, Puglia, Sardinia) who gave birth in a hospital between 1 April 2016, and 31 March 2018. The study excluded voluntary abortions and miscarriages with gestational age fewer than 180 days and birthweight less than 500 g, as this information was not recorded in the CeDAP database.

For each delivery, the initiation date of pregnancy was estimated by calculating the difference between the date of birth and the gestational age at birth, expressed in days (computed by multiplying the number of weeks of amenorrhea by 7 days). The study identified three distinct time windows:

- Pre-pregnancy period, defined as the three trimesters before the last menstrual period (LMP) date (273 days before LMP date).
- Pregnancy period, which included the I trimester of pregnancy (1st TP) defined as the period between 0 (LMP date = start date of pregnancy) and day 91 following the start date of pregnancy; the II trimester of pregnancy (2nd TP), defined as the period between day 92 and day 189 from the start date of pregnancy (or date of birth if the birth occurred during the 2nd TP, which is within 27 weeks of gestation); the III trimester of pregnancy (3rd TP) defined as the period between day 190 from the start of pregnancy and the date of birth.
- Post-pregnancy period defined as the three trimesters after the date of birth (273 days following the date of birth).

The prevalence of drug use was defined as the percentage of women with at least one drug prescription during the period considered (one of the three trimesters). The overall prevalence of antihypertensive use in the entire cohort of women was examined before, during, and after pregnancy.

The classification of drug use prevalence as “prevalent” occurred when the drug was prescribed before conception (i.e. 14 days after last menstrual period), while it was termed “incident” if there was a new prescription during pregnancy.

The analysis included an examination of the transitions between different subgroups of antihypertensive drugs within users. Specifically, the Sankey Diagram was employed to visually represent the patterns of drug use during different pregnancy trimesters. In this graphical flow diagram, the width of the arrows corresponded to the flow rate, enabling the illustration of the percentage of women who either discontinued treatments or shifted from not recommended drugs to recommended treatments during pregnancy.

Additionally, the study delved into regional variations in antihypertensive prescriptions. Statistical analyses were carried out using SAS and R.

The switch between different drug’s subgroups within antihypertensive user was analyzed. In particular, pattern of drug use in the different pregnancy trimesters was represented through the Sankey Diagram. In this flow diagram the width of the arrows is proportional to the flow rate and it allows to display the percentage of women who interrupted treatments or switched from not recommended drugs to treatments of choice in pregnancy. Moreover, the differences of antihypertensive prescriptions among regions were analyzed. Statistical analyses were performed using SAS and R.

## Results

The dataset encompasses a cohort of 449,012 women, aged between 15 and 49 years, who gave birth during the period from 1 April 2016, to 31 March 2018, across the eight participating regions. This cohort represents 59% of all deliveries in Italy during the specified timeframe. Table 1 describes the overall distribution of women prescribed with antihypertensive drugs, from the three trimesters preceding the conception to the three trimesters after child delivery. Multiple pregnancies were 1.8% of the total.

**TABLE 1:** Study cohort characteristics (n = 449,012).

	n	%
Age group		
≤24	33 651	7.5
25−29	92 333	20.6
30−34	154 588	34.4
35−39	124 680	27.8
≥ 40	43 760	9.7
Of which ≥ 45	3 438	7.9
Nationality		
Italian	358 467	79.8
Foreign	88 629	19.8
High income countries	86 159	97.7
Low income countries	2 470	2.3
Level of education		
None/primary school	106 759	23.8
Secondary school	200 618	44.7
Bachelor degree/post-bachelor degree	139 559	31.1
Missing	2 076	0.5
Occupational status		
Employed	284 069	63.3
Unemployed/Looking for first job	54 492	12.1
Housewife	98 450	21.9
Other	7 210	1.6
Missing	4 791	1.1
Previous delivery		
No	227 525	50.7
Yes	221 487	49.3
Of which cesarean section	59 782	27.0
Previous spontaneous abortions*		
0	360 619	80.3
1	65 997	14.7
2	22 396	5.0
Gestational age		
Preterm delivery (<37 weeks)	30 774	6.9
Term delivery (37–41 weeks)	415 366	92.5
Post-term delivery (>41 weeks)	2 872	0.6
Parity		
1	440 765	98.2
2+	8 247	1.8
Invasive prenatal diagnosis		
None	394 785	88.1
Chorionic villus sampling	20 435	4.6
Amniocentesis	31 423	7.0
Other invasive test	1 433	0.3
Medically assisted procreation**		
no/not classified	360.558	97,0
Yes	11.233	3,0
Cesarean section		
No	312.785	69,7
Yes	136.227	30,3

*Lazio took in account number of voluntary and spontaneous abortion.

**Data from Lazio and Umbria are not included.

**FIGURE 1:**
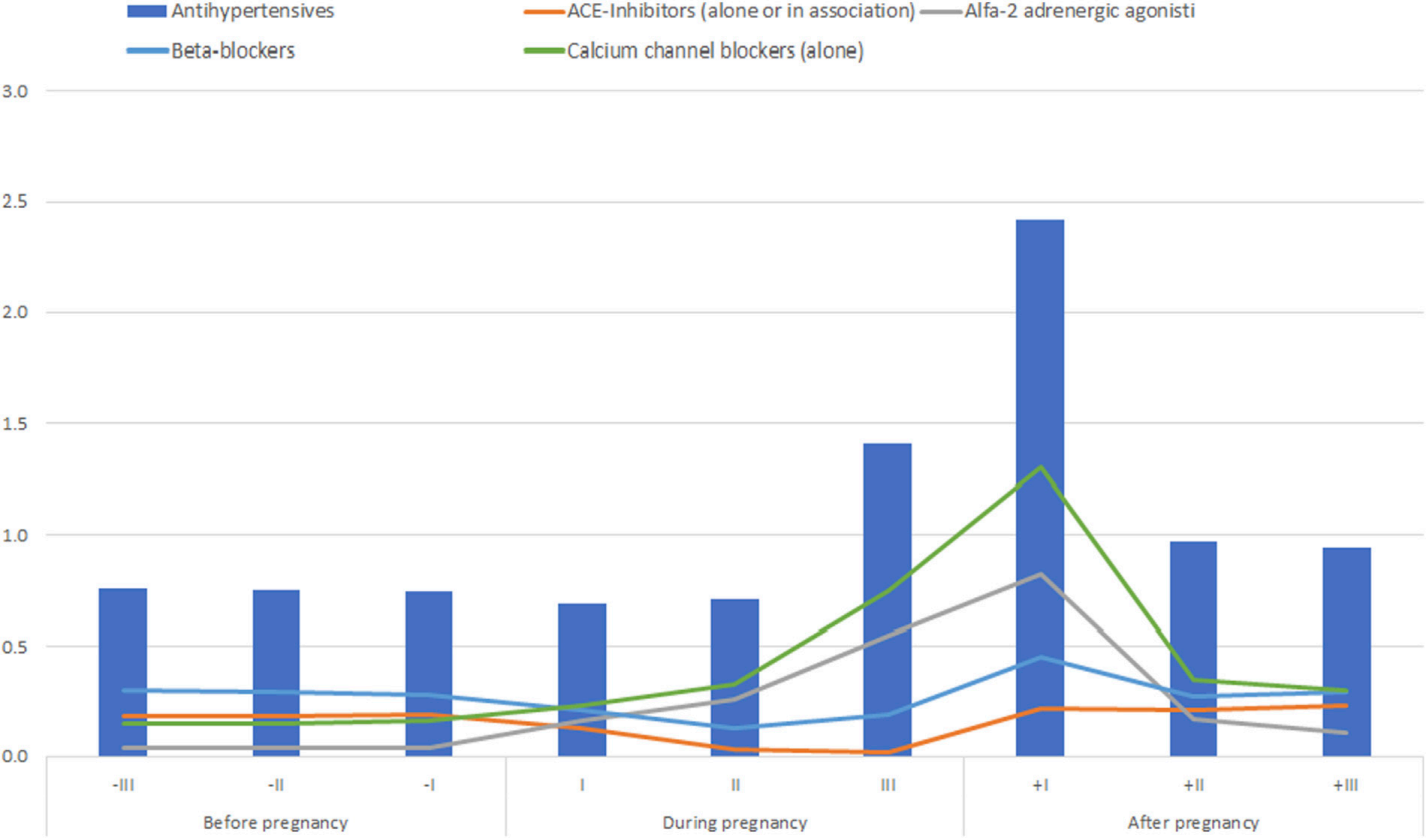
Prevalence of use (%) of antihypertensives in the trimesters before, during and after pregnancy.

The prevalence of prescription of antihypertensive drugs in the pre-conceptional period was 1.2%, raised to 2.0% during pregnancy and to 2.9% in the postpartum period, which was almost stable in the three pre-conceptional trimesters (0.74%–0.76%), slightly decreased in the first trimester of pregnancy (0.69%), then raised to 0.71% in the second trimester and to 1.41% in the third one (Figure 1; Supplementary Table S1). The peak of drug prescription (2.4%) was observed in the first trimester after delivery, then the prevalence gradually decreased in the next two trimesters, even though it did not return to the pre-conceptional levels.

Calcium channel blockers are the most prescribed drugs in all pregnancy trimesters and in the first trimester after delivery: the prescription prevalence ranged from 0.23% in the first trimester to 0.33% in the second trimester, peaking in the third one with a prevalence of 0.75%.

Alfa-2-adrenergic receptor agonists were the second most prescribed antihypertensive drug during pregnancy with a prevalence of 0.16%, 0.26% and 0.55% in each trimester.

Beta-blockers were the most prescribed drugs in the trimesters before pregnancy with a prevalence of 0.30%, 0.29% and 0.28% respectively. The prescription of this antihypertensive class decreased during pregnancy with a prevalence ranging from 0.13% to 0.21%. The prevalence of prescription of drugs contraindicated in pregnancy, such as angiotensin-converting enzyme (ACE) inhibitors angiotensin receptor blockers (ARBs) alone or in association, was below 0.5% in all pregnancy trimesters (Figure 1).

In the preconceptional trimester, among patients with at least one drug prescription, the most prescribed antihypertensive drugs were ACE-inhibitors or ARBS (either alone or in association) with a prevalence of 53.4% (1766/3309). This was followed by beta-blockers at 27.4% (906/3309), calcium-channel blockers at 15% (483/3309), and alpha-2 adrenergic receptor agonists at 3.7% (121/3309). In the first trimester of pregnancy the most frequently prescribed drugs were, equally, calcium-channel blockers and beta blockers, each accounting for both 23.1% (718/3104). These were followed by alfa-2 adrenergic receptor agonist at 14.8% (459/3104). Not recommended drugs were prescribed at a rate of 38.3% (1189/3104). Lastly, in the third trimester of pregnancy, calcium-channel blockers were the most prescribed drugs, accounting for 50.9% (3224/6333). This was followed by alpha-2 adrenergic receptor agonists at 31% (1964 out of 6333), beta-blockers at 12.7% (802/6333), and drugs not recommended for pregnancy at 5.2% (329/6333).

In a total of 12,647 prescriptions of antihypertensive drugs, 1,902 (0.15%) were inappropriate–including ACE-inhibitors, ARBs, direct renin inhibitors and diuretics–the majority of which [63.6% (1205/1902)] in the first pregnancy trimester.

At the beginning of pregnancy, the 18.8% of prevalent users (i.e., chronic or pre-existent hypertension using drug in the trimester before pregnancy) suspended the antihypertensive treatment, whereas the number of new users (i.e., pregnancy-related disease) gradually increased during pregnancy, reaching a peak in the third pregnancy trimester.

**FIGURE 2:**
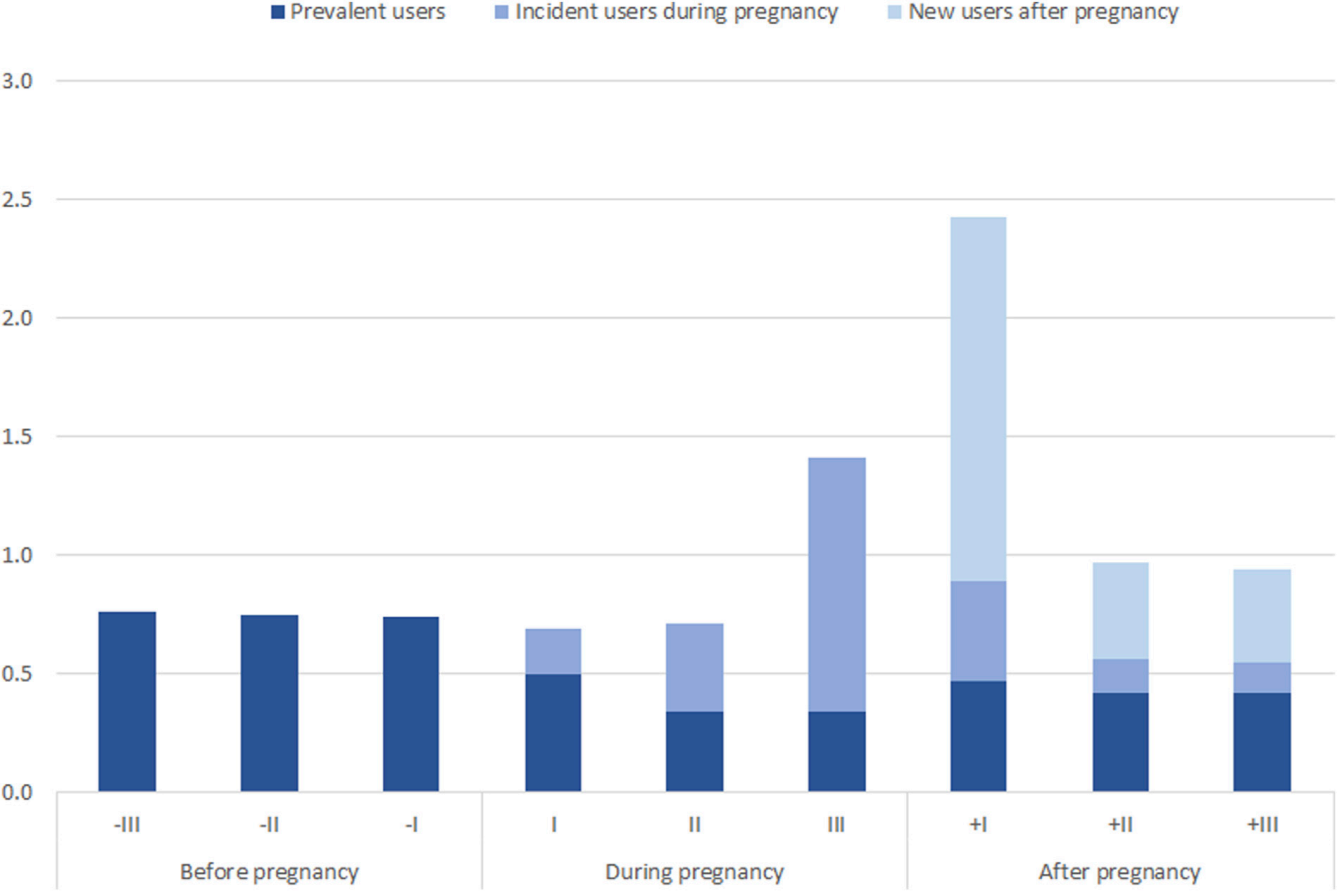
Prevalence of use (%) of antihypertensives in new users.

In the first postpartum trimester the highest prevalence of new users was observed (Supplementary Table S2; Figure 2).

**FIGURE 3:**
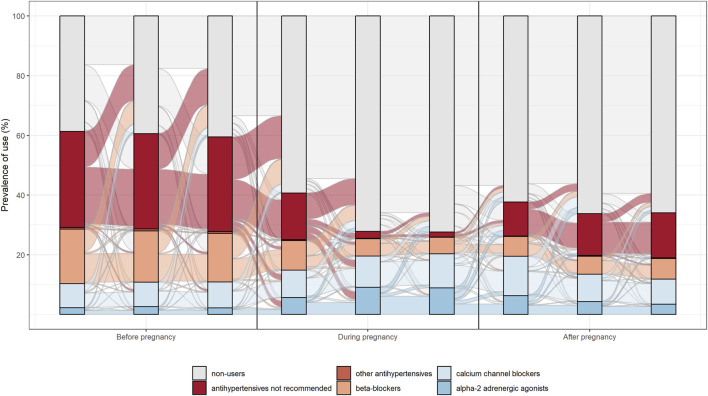
Pattern of prescription of antihypertensive drugs.

We saw that only a small percentage of women switched from a contraindicated drug to a drug compatible with pregnancy, compared with number of women who interrupted the drug treatment at the beginning of pregnancy, being therefore untreated (Figure 3).

**FIGURE 4:**
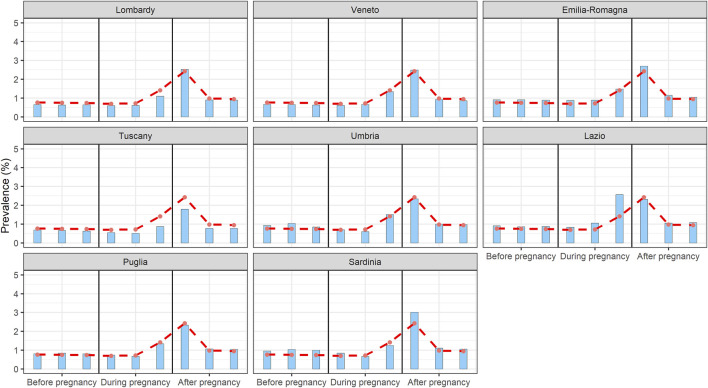
Prevalence of antihypertensives prescription in the different Italian regions in the trimesters before, during and after pregnancy.

The analysis of drug prescription by Italian region included in the study showed little variability ranging from 1.4% to 1.62% before, 1.29%–3.32% during, and 2.21%–3.43% after pregnancy respectively. Only in Lazio region we observed a higher prevalence of antihypertensive prescriptions in pregnancy (3.32% overall in pregnancy, and in particular in the third trimester; Umbria, the second more prescribing region, only reached the 2.07%) (Figure 4). This data were confirmed after adjusting for maternal age (considering the mean of the regional maternal age, data available upon request).

## Discussion

The prescribing profile of antihypertensive drugs in the Italian MoM-Net cohort seemed to be coherent with drugs compatible with pregnancy (Abalos et al., 2018). The prescriptions of antihypertensive drugs contraindicated in pregnancy, although contained, could be further improved with the planning of pregnancy, recommended in the presence of chronic diseases. In fact, the most prescribed drug class in all pregnancy trimesters were calcium channel blockers, while the overall prescription rate of contraindicated antihypertensives, such as ACE inhibitors and ARBs alone or in combination, was low (0.5% overall, 15% of the antihypertensive drugs) even if higher respect to 0.12% (5% of antihypertensive drugs) reported in France (Duhalde et al., 2011).

The analysis of the prescription pattern of the different classes of antihypertensive drugs across all trimesters showed a withdrawal from the antihypertensive therapy for prevalent users at the beginning of pregnancy (i.e., women affected by chronic or pre-existent hypertension), whereas the number of new users (i.e., women with pregnancy-related disease, such as gestational hypertension or preeclampsia) gradually increased during pregnancy period, reaching a peak in the third trimester. Women who interrupted the antihypertensive therapy during pregnancy were mostly prescribed with non-recommended drugs and the number of women who switched from a non-recommended drug to a recommended drug was limited, suggesting that women who interrupted the non-recommended drug treatment remained effectively without an antihypertensive therapy during pregnancy. The appropriateness of drug prescription before conception seemed to be therefore questionable.

The appropriateness of maintaining antihypertensive drug treatment in hypertensive women during pregnancy has long been a subject of uncertainty. In 2018, a Cochrane systematic review failed to provide clear guidance on the justification of antihypertensive drug treatment for mild to moderate hypertension during pregnancy (National Institute for Health and Care Excellence UK, 2019). Additionally, the CHIPS trial did not fully clarify the impact of less-tight versus tight control of hypertension on pregnancy complications (Magee et al., 2016). The existing ambiguity underscores the need for further research and understanding in this area of maternal healthcare.

Recently, the CHAP Trial Group proposed that in women with mild chronic hypertension, aiming for a blood pressure target of less than 140/90 mmHg resulted in improved pregnancy outcomes compared to a strategy of reserving treatment exclusively for severe hypertension. Notably, this approach did not elevate the risk of small-for-gestational-age birth weight (Tita et al., 2022).

In the CHIPS trial the most prescribed drugs were labetalol (66.9% and 67.3% in the less-tight and in the tight control groups, respectively), followed by alpha-methyldopa (43.5% vs. 40.3%) and nifedipine (31.8% vs. 30.1%). Similar trends were observed in the CHAP trial.

In our study we observed a less frequent prescription of labetalol, with nifedipine and alfa-methyldopa being preferred. Additionally, we observed that among incident users the most prescribed drugs were calcium-channel blockers, a finding that slightly differs from the CHIPS and CHAP trial.

In international guidelines, the first-line therapy involves either labetalol or nifedipine (Scott et al., 2022). These guidelines commonly do not provide specific recommendations regarding tailoring drug therapy based on ethnicity or on pathophysiology. An ongoing randomized controlled trial is currently underway to provide further evidence on selecting the optimal hypertensive treatment between calcium-channel blockers and beta-blockers, results are expected in 2025 (Ashworth et al., 2023).

A recent prospective multicenter observational study showed promising results when the first-line antihypertensive agent was tailored to the correct maternal haemodynamic profile (di Pasquo et al., 2024).

In our study, the suspension of contraindicated drugs without the introduction of alternative therapy could be linked to the lower blood pressure values observed during pregnancy. Alternatively, it raises questions about the appropriateness of prior prescriptions in these cases. A comprehensive individual clinical analysis, considering blood pressure values and outcomes, is necessary to better address this question and understand the implications of discontinuing contraindicated drugs during pregnancy. Interestingly our study offers for the first time a real picture of the antihypertensive drugs use in the fertile population in Italy, and indirectly, even if with some limitations, the prevalence of chronic hypertension.

The prescription pattern in new users in pregnancy was compatible with the observed prevalence of gestational hypertension and preeclampsia and the most prescribed drugs are those compatible with pregnancy (Khan et al., 2006; Lean et al., 2017; Ukah et al., 2018). This could be interpreted as a timely diagnosis and an appropriate treatment of hypertension in pregnancy, even though there is still a small percentage of contraindicated drug prescriptions. Hypertensive disorders can also affect the puerperium, even in women not previously diagnosed with hypertension (Magee and von Dadelszen, 2013) and our data reflect this phenomenon.

The prescribing pattern observed in new pregnant users appeared to align with the onset of gestational hypertension or preeclampsia. This was evident from the preferential use of recommended drug classes such as calcium channel blockers and alpha-2 adrenergic agonists, indicating a timely management approach when hypertension arose during pregnancy. However, it is noteworthy that a small percentage of non-recommended drug prescriptions persisted in all trimesters of pregnancy. This highlights the need for heightened attention to the assessment of prescribing appropriateness, emphasizing the importance of ensuring that the medications prescribed align with established guidelines, especially during pregnancy and breastfeeding.

Our analysis showed only little regional variability, suggesting appropriate therapeutic choices about the antihypertensive drugs throughout Italy. Lazio region was the only exception, with a prevalence of 2.6% of antihypertensive prescriptions in the third trimester, probably due to differences in treatment choices or in the gestational hypertension/preeclampsia prevalence (Figure 4). We should note otherwise that data on regional prevalence of hypertension are not available.

Our data could promote evidence-based public interventions in maternal health. The prescription pattern of contraindicated antihypertensives could be improved with preconception visit/preconception control, being it mostly recommended in women with chronic pathologies. Women diagnosed with chronic hypertension planning a pregnancy should be evaluated in the pre-conceptional period to receive the most effective, appropriate, and safest treatment for the mother and the fetus, as it is recommended in the international guidelines for the management of hypertension in pregnancy (National Institute for Health and Care Excellence UK, 2019).

The strength of our study lies in the comprehensive medication prescription data during pregnancy obtained from eight diverse Italian regions, collectively representing all geographical areas. These regions, including Emilia-Romagna in the North, Lazio in the Centre, and Puglia in the South, contribute to the study’s robust representation. As far as our knowledge extends, this study stands as the largest and most representative population-based analysis of antihypertensive drug prescriptions during pregnancy in Italy.

However, a limitation of the study is that our administrative databases lack information on drug use in pregnancies that ended in miscarriage or induced abortion. Additionally, there is an absence of data on therapeutic indications and pregnancy outcomes associated with drug prescribing. Consequently, we were unable to delve more deeply into the patterns of medication use during pregnancy or explore the relationship between drug prescriptions and specific pregnancy outcomes. What is missing in our study is the correlation between clinical characteristics before pregnancy, the severity of the hypertensive disorder and comorbidities or the effect of the prescriptions made. Moreover, we cannot capture with our database the hospital use of drugs, mostly related to severe cases and preeclampsia, often leading to drug use after delivery and after discharge and captured in the records after pregnancy.

Another limitation to our study is that we only included women who delivered in hospital. This may potentially result in an overestimation of the prevalence of anti-hypertensive drug use. It is important to note, however, that home births in Italy constitute only a minimal proportion, accounting for 0.1% of deliveries, and typically involve low-risk pregnancies. While this limitation should be considered, the impact on the overall findings is likely mitigated by the rarity of home births in the context of the broader study population.

## Conclusions

The prescription pattern of antihypertensive drugs in the Italian MoM-Net cohort, which is the largest and most representative population-based study on medication prescription during pregnancy in Italy, appears to align with medications compatible with pregnancy. Despite the limitations in available information, the conducted analyses offer an updated and comprehensive overview of antihypertensive drug prescribing in Italian pregnant women. These findings contribute to the identification of critical aspects in the management of hypertensive disorders during pregnancy in Italy. The descriptive studies conducted within the MoM-Net group and coordinated nationally have the potential to enhance Italian clinical practice by informing treatment choices during pregnancy. Additionally, they can serve as a catalyst for interventions aimed at reducing intra-regional and inter-regional variability in prescription patterns. This collaborative effort holds promise for improving the consistency and quality of care for pregnant women with hypertensive conditions across different regions of Italy.

Further studies are needed to determine the optimal antihypertensive drugs regimen during pregnancy since our data do not allow to draw definitive recommendations.

## Data Availability

The raw data supporting the conclusions of this article will be made available by the authors, without undue reservation.

## Group member of MoM-Net group

Filomena Fortinguerra, Serena Perna, Francesco Trotta, (Italian Medicines Agency, Rome); Renata Bortolus, Giovanni Rezza (Directorate General for Preventive Health - Office 9, Ministry of Health, Rome); Paola D’Aloja, Serena Donati (National Centre for Disease Prevention and Health Promotion, Istituto Superiore di Sanità, Rome); Roberto Da Cas (Pharmacoepidemiology Unit, National Centre for Drug Research and Evaluation, Istituto Superiore di Sanità, Rome); Antonio Addis, Valeria Belleudi, Marina Davoli, Lorella Lombardozzi, Francesca Romana Poggi (Department of Epidemiology, Lazio Regional Health Service, ASL Roma 1, Rome); Antonio Clavenna (Laboratory for Pharmacoepidemiology, Department of Public Health, IRCCS – Istituto di Ricerche Farmacologiche Mario Negri, Milan, Italy ); Anna Locatelli (Obstetrics and Gynecology Unit, School of Medicine and Surgery, University of Milano Bicocca, Milan); Ida Fortino, Arianna Mazzone, Simone Schiatti, Martina Zanforlini (Lombardy Region); Paola Deambrosis, Silvia Manea, Laura Salmaso, Giovanna Scroccaro (Veneto Region), Anna Maria Marata, Aurora Puccini, Valentina Solfrini (Emilia-Romagna Region); Francesco Attanasio, Rosa Gini (Tuscany Region); Marcello De Giorgi, David Franchini, Mariangela Rossi (Umbria Region); Vito Montanaro, Paolo Stella (Apulia Region); Paolo Carta, Donatella Garau, Stefano Ledda, Enrico Serra (Sardinia Region).
