# Supplementary material for: Antihypertensive therapy during pregnancy: the prescription pattern in Italy
Source: Front Pharmacol. 2024 Aug 30;15:1370797. doi: 10.3389/fphar.2024.1370797 (PMC11393683; doi:10.3389/fphar.2024.1370797)
Supplement: Supplementary file 1 [file Table1.DOCX]

SUPPLEMENTARY MATERIALS

**SUPPLEMENTARY MATERIAL**

|  | **BEFORE PREGNANCY** | | | | | | | **DURING PREGNANCY** | | | | | | | | **AFTER PREGNANCY** | | | | | | | |
| --- | --- | --- | --- | --- | --- | --- | --- | --- | --- | --- | --- | --- | --- | --- | --- | --- | --- | --- | --- | --- | --- | --- | --- |
|  | **-III** | | **-II** | | **-I** | | | **I** | | **II** | | | **III** | | | **+I** | | **+II** | | | **+III** | | |
|  | n | % | n | % | | n | % | n | % | | n | % | | n | % | n | % | | n | % | | n | % |
| **Antihypertensives** | **3.409** | **0,76** | **3.370** | **0,75** | | **3.309** | **0,74** | **3.104** | **0,69** | | **3.210** | **0,71** | | **6.333** | **1,41** | **10.885** | **2,42** | | **4.375** | **0,97** | | **4.233** | **0,94** |
| ACE-inhibitors (alone or in association) | 802 | 0,18 | 816 | 0,18 | | 833 | 0,19 | 564 | 0,13 | | 140 | 0,03 | | 103 | 0,02 | 979 | 0,22 | | 957 | 0,21 | | 1.012 | 0,23 |
| Alfa-2 adrenergic receptor agonists | 192 | 0,04 | 202 | 0,04 | | 185 | 0,04 | 728 | 0,16 | | 1.160 | 0,26 | | 2.457 | 0,55 | 3.695 | 0,82 | | 752 | 0,17 | | 510 | 0,11 |
| Angiotensin receptor blockers (alone or association) | 573 | 0,13 | 539 | 0,12 | | 538 | 0,12 | 377 | 0,08 | | 114 | 0,03 | | 105 | 0,02 | 478 | 0,11 | | 562 | 0,13 | | 620 | 0,14 |
| Beta-blockers (alone or in association) | 1.337 | 0,30 | 1.281 | 0,29 | | 1.259 | 0,28 | 957 | 0,21 | | 602 | 0,13 | | 848 | 0,19 | 2.006 | 0,45 | | 1.202 | 0,27 | | 1.308 | 0,29 |
| Calcium-Channel Blockers (alone) | 671 | 0,15 | 681 | 0,15 | | 714 | 0,16 | 1.011 | 0,23 | | 1.484 | 0,33 | | 3.348 | 0,75 | 5.830 | 1,30 | | 1.585 | 0,35 | | 1.330 | 0,30 |
| Diuretics | 406 | 0,09 | 417 | 0,09 | | 378 | 0,08 | 235 | 0,05 | | 90 | 0,02 | | 124 | 0,03 | 824 | 0,18 | | 443 | 0,10 | | 402 | 0,09 |
| Peripheric adrenergic Alfa-receptor blockers | 46 | 0,01 | 57 | 0,01 | | 54 | 0,01 | 28 | 0,01 | | 11 | 0,00 | | 10 | 0,00 | 56 | 0,01 | | 52 | 0,01 | | 51 | 0,01 |
| Aliskiren (alone or in association) | 4 | 0,00 | 4 | 0,00 | | 3 | 0,00 | 1 | 0,00 | | 0 | 0,00 | | 0 | 0,00 | 0 | 0,00 | | 0 | 0,00 | | 1 | 0,00 |

**Table 1s.** Women with at least a prescription of an antihypertensive drug in the trimesters before, during or after pregnancy

|  | **BEFORE PREGNANCY** | | | | | | **DURING PREGNANCY** | | | | | | **AFTER PREGNANCY** | | | | | |
| --- | --- | --- | --- | --- | --- | --- | --- | --- | --- | --- | --- | --- | --- | --- | --- | --- | --- | --- |
|  | **-III** | | **-II** | | **-I** | | **I** | | **II** | | **III** | | **+I** | | **+II** | | **+III** | |
|  | n | % | n | % | n | % | n | % | n | % | n | % | n | % | n | % | n | % |
| **Prevalent users**  **N=5.560** |  |  |  |  |  |  |  |  |  |  |  |  |  |  |  |  |  |  |
| Not recommended drugs ^ | 1.788 | 32,2 | 1.777 | 32,0 | 1.766 | 31,8 | 871 | 15,7 | 132 | 2,4 | 95 | 1,7 | 634 | 11,4 | 780 | 14,0 | 840 | 15,1 |
| Other (antihypertensive) | 31 | 0,6 | 37 | 0,7 | 33 | 0,6 | 17 | 0,3 | 5 | 0,1 | 3 | 0,1 | 9 | 0,2 | 13 | 0,2 | 10 | 0,2 |
| Beta-blockers | 1.015 | 18,3 | 954 | 17,2 | 906 | 16,3 | 551 | 9,9 | 324 | 5,8 | 307 | 5,5 | 370 | 6,7 | 337 | 6,1 | 386 | 6,9 |
| Calcium-Channel Blockers | 448 | 8,1 | 458 | 8,2 | 483 | 8,7 | 510 | 9,2 | 582 | 10,5 | 635 | 11,4 | 731 | 13,1 | 508 | 9,1 | 466 | 8,4 |
| Alfa-2 adrenergic receptor agonists | 127 | 2,3 | 144 | 2,6 | 121 | 2,2 | 315 | 5,7 | 506 | 9,1 | 496 | 8,9 | 352 | 6,3 | 241 | 4,3 | 191 | 3,4 |
| Non users | 2.151 | 38,7 | 2.190 | 39,4 | 2.251 | 40,5 | 3.296 | 59,3 | 4.011 | 72,1 | 4.024 | 72,4 | 3.464 | 62,3 | 3.681 | 66,2 | 3.667 | 66,0 |
| **New users in pregnancy** **N=6.067** |  |  |  |  |  |  |  |  |  |  |  |  |  |  |  |  |  |  |
| Not recommended drugs ^ | 0 | 0,0 | 0 | 0,0 | 0 | 0,0 | 318 | 5,2 | 212 | 3,5 | 234 | 3,9 | 260 | 4,3 | 231 | 3,8 | 245 | 4,0 |
| Other (antihypertensive) | 0 | 0,0 | 0 | 0,0 | 0 | 0,0 | 3 | 0,0 | 5 | 0,1 | 7 | 0,1 | 5 | 0,1 | 4 | 0,1 | 5 | 0,1 |
| Beta-blockers | 0 | 0,0 | 0 | 0,0 | 0 | 0,0 | 167 | 2,8 | 230 | 3,8 | 495 | 8,2 | 293 | 4,8 | 103 | 1,7 | 98 | 1,6 |
| Calcium-Channel Blockers | 0 | 0,0 | 0 | 0,0 | 0 | 0,0 | 208 | 3,4 | 768 | 12,7 | 2.589 | 42,7 | 815 | 13,4 | 209 | 3,4 | 168 | 2,8 |
| Alfa-2 adrenergic receptor agonists | 0 | 0,0 | 0 | 0,0 | 0 | 0,0 | 144 | 2,4 | 428 | 7,1 | 1.472 | 24,3 | 504 | 8,3 | 102 | 1,7 | 63 | 1,0 |
| Non users | 6.067 | 100,0 | 6.067 | 100,0 | 6.067 | 100,0 | 5.227 | 86,2 | 4.406 | 72,6 | 1.270 | 20,9 | 4.190 | 69,1 | 5.418 | 89,3 | 5.488 | 90,5 |

^ *ACE-inhibitors and ARBs alone or in association*

**Table 2s.** Pattern of prescription of antihypertensives in the trimesters before, during and after pregnancy
